# Evaluation of humoral immune response and milk antibody transfer in calves and lactating cows vaccinated with inactivated H5 avian influenza vaccine

**DOI:** 10.1038/s41598-025-87831-w

**Published:** 2025-02-07

**Authors:** Mohamed Samy Abousenna, Nermeen G. Shafik, Mahmoud M. Abotaleb

**Affiliations:** Central Laboratory for Evaluation of Veterinary Biologics, Agricultural Research Center, P.O. Box 131, Cairo, 11381 Egypt

**Keywords:** Highly pathogenic avian influenza, AIV vaccine, Vaccine efficacy, HI, AIV in cattle, AIV outbreak, Influenza virus, Inactivated vaccines, Inactivated vaccines

## Abstract

The detection of Highly Pathogenic Avian Influenza (HPAI) A(H5N1) in dairy cattle in the United States has raised concerns about human exposure. This study evaluated the efficacy of various doses of an inactivated H5 AI vaccine in cattle and assessed antibody transfer in milk against a recent bovine isolate of HPAI A(H5N1, clade 2.3.4.4b). Calves were inoculated with different vaccine doses, while lactating cows received the vaccine four weeks later. The humoral immune response was measured using the Hemagglutination Inhibition (HI) test and ELISA. Results showed a dose-dependent immune response, with higher doses producing stronger and more sustained antibody levels. Group 1 maintained a stable HI titer of 6 log_2_, while Groups 2, 3, and 4 peaked at 8, 9, and 9 log_2_, respectively, by the fourth week post-vaccination. Milk antibody transfer was observed, with strong positive responses in milk samples by the second week post-vaccination. The ID Screen ELISA demonstrated higher sensitivity for detecting antibodies in milk compared to serum. The immune response to the AI vaccine differed from responses to other vaccines used in cattle such as Foot and Mouth Disease Virus (FMDV) and Lumpy Skin Disease Virus (LSDV), indicating the need for optimizing vaccine dosage and formulation, including adjuvant and antigen content. Future research should extend the monitoring period, increase sample sizes, and explore different vaccine formulations to develop effective vaccination strategies for cattle. These findings highlight the potential for using inactivated H5 AI vaccines in cattle to enhance immune protection and facilitate antibody transfer through milk.

## Introduction

Avian influenza viruses (AIVs) pose significant obstacles to worldwide public health systems due to their extensive prevalence and substantial rates of mortality^1^, AIVs are divided into two classifications depending on their ability to cause illness in chickens as assessed by the intravenous pathogenicity index (IVPI) test: highly pathogenic avian influenza viruses (HPAIV) and low pathogenic avian influenza viruses (LPAIV)^2,3^.

Highly pathogenic avian influenza (HPAI) viruses are a global threat to both wild birds and poultry, with particular concern surrounding HPAI H5N1 viruses due to their frequent transmission to mammals^4^. The Eurasian strain of H5N1 (clade 2.3.4.4b) was detcted in North America in late 2021, triggering an outbreak that persisted into 2024. Cases of transmission and fatalities stemming from this clade have been observed in various terrestrial and marine mammal species within the United States^5–7^.

A bovine syndrome emerged in February 2024, affecting lactating dairy cattle within the Texas panhandle region. Initial presentation included non-specific illness, decreased feed intake and rumination, and a marked decline in milk production. Notably, milk from affected cows displayed a thickened, creamy yellow appearance reminiscent of colostrum. The syndrome exhibited a wave-like pattern within affected farms, with peak incidence occurring 4–6 days after initial presentation, followed by a decline within 10–14 days. Most animals transitioned back to regular milking routines thereafter. Clinical signs predominantly manifested in multiparous cows during mid to late lactation. Morbidity rates hovered around 10–15%, with minimal mortality observed. Initial diagnostic workup, including blood, urine, feces, milk, nasal swab samples, and postmortem tissues, yielded no definitive cause for the reduced milk production. Milk cultures were frequently negative, while serum chemistry revealed mildly elevated levels of aspartate aminotransferase, gamma-glutamyl transferase, creatinine kinase, and bilirubin. Complete blood count results varied, with some animals exhibiting anemia and leukocytopenia.

The geographical spread widened in early March 2024, with similar clinical presentations reported in dairy cattle of southwestern Kansas and northeastern New Mexico. Notably, this period also coincided with reports of wild bird and domestic cat mortalities within affected Texas panhandle sites. In Texas, feline deaths were documented on dairy farms where raw colostrum and milk from sick cows were fed to domestic cats housed within hospital parlors. Antemortem clinical signs in affected cats included lethargy, stiff body movements, ataxia, blindness, circling behavior, and copious oculonasal discharge. Neurological examinations revealed absent menace responses and pupillary light reflexes, with only weak blink responses remaining.

On March 21, 2024, the Iowa State University Veterinary Diagnostic Laboratory (ISUVDL; Ames, IA, USA) received milk, serum, fresh, and fixed tissue samples from affected cattle in Texas dairies, along with tissues from two deceased cats originating from an affected Texas farm. The following day, similar sample sets arrived from affected Kansas dairies. Subsequent testing via screening PCR identified influenza A virus (IAV) in milk and tissue samples from cattle and tissues from the cats. Confirmation and characterization by the US Department of Agriculture National Veterinary Services Laboratory revealed the causative agent to be highly pathogenic avian influenza H5N1 virus. This detection prompted an initial press release by the US Department of Agriculture Animal and Plant Health Inspection Service on March 25, 2024, confirming the presence of HPAI virus in dairy cattle^8,9^.

Genetic analysis of HPAI H5N1 strains isolated from Egyptian avian hosts revealed close homology to those circulating throughout Europe, North America, Asia, and Africa during the 2021–2022 season. Whole genome sequencing identified markers associated with mammalian adaptation and increased virulence across various gene segments, mirroring patterns observed in European and African HPAI H5N1 strains. The presence of clade 2.3.4.4b HPAI H5N1 in Egyptian wild birds highlights the potential for spillover events into domestic poultry and cattle populations, posing a subsequent risk to human health^10^.

The extensive circulation of HPAI A(H5N1) in avian populations, poultry farms, and specific mammalian species, including bovines, presents a potential avenue for increased human exposure. The identification of severe human illness caused by HPAI H5N1 clade 2.3.4.4b in Ecuador and Chile underscores concerns regarding the pandemic potential of certain HPAI strains^11,12^. This scenario suggests a possibility of heightened sporadic human infections arising from zoonotic transmission, even if the inherent transmissibility of these viruses from birds to humans remains unchanged. While the CDC maintains a low-risk assessment for the general public regarding avian influenza viruses, individuals with occupational or recreational exposure to infected birds and animals, encompassing cows, demonstrably face a heightened risk of contracting HPAI A(H5N1)^13^.

Current management strategies for avian influenza (AIV) infections encompass a multi-pronged approach. The primary method often involves the culling of infected poultry populations to prevent further viral spread within flocks and neighboring farms^14^. Vaccination programs also play a critical role in AIV control. Conventional inactivated vaccines offer a preventative measure, but their effectiveness hinges on close genetic and antigenic matching with circulating viral strains. However, this approach can be hampered by the rapid antigenic evolution of AIVs. Therefore, ongoing research and development efforts focus on novel vaccines with broader protection against diverse AIV strains. Additionally, biosecurity measures, including strict hygiene protocols, movement restrictions within and between farms, and proper disposal of carcasses and waste materials, are crucial for preventing the introduction and dissemination of AIV. Early detection through surveillance programs allows for prompt intervention and implementation of control measures to minimize the impact of outbreaks^15,16^.

The current manuscript aims to optimize the dosage of an inactivated H5 avian influenza virus vaccine in cattle by evaluating the dose-dependent humoral immune response in calves and assessing antibody transfer through milk in lactating cows. This study seeks to identify the most effective dose for generating a strong and sustained immune response, with an emphasis on enhancing vaccine efficacy and antibody transfer potential.

## Materials and methods

### Virus

The HPAI H5N1 virus, specifically the A/ibis/Egypt/RLQP-229 S/2022(H5N1) strain belonging to clade 2.3.4.4b, was isolated and sequenced (ACCESSION OP491851) at the Animal Health Research Institute in Egypt. This virus was then provided to the Central Laboratory for Evaluation of Veterinary Biologics (CLEVB) for the purpose of assessing the efficacy of the existing inactivated H5 Avian Influenza vaccine. For this assessment, the virus was treated with Binary Ethylenimine (BEI) to be used as a Hemagglutination Inhibition (HI) antigen.

### Vaccine

The H5 Avian Influenza vaccine batch available for this experiment (*n* = 1) had previously been assessed by CLEVB, yielding satisfactory results. This is an oil adjuvant inactivated reassorting avian influenza vaccine prepared from two strains:

H5N6 strain Re13 (A/duck/Fujian/S1424/2020) from clade 2.3.4.4 h.

H5N8 strain Re14 (A/whooper swan/Shanxi/4 − 1/2020) from clade 2.3.4.4b.

#### Evaluation of humoral immune response

##### Animal groups and experimental design

This study was conducted at the Central Laboratory for Evaluation of Veterinary Biologics (CLEVB) at the Agricultural Research Center (ARC), with approval from the ARC Institutional Animal Care and Use Committee (ARC-IACUC). A total of twenty-two native Egyptian calves Baladi (Menufi breed aged 6 to 8 months) were included in the study. For experimental purposes, these animals were categorized into five groups, housed separately within the CLEVB animal facility, and assigned different vaccine doses to evaluate the dose-dependent immune response.

All animals were initially screened for antibodies against Influenza A virus using the ID Screen ELISA Influenza A Antibody Competition Multi-Species test. Only seronegative calves were included in the study to ensure accurate assessment of the vaccine’s efficacy.

Group Allocation and Vaccine Dosage.

Calves:

Group 1: Five calves received a subcutaneous (S/C) injection of four field doses (2 ml each) of the H5 AI vaccine, following the manufacturer’s instructions for the evaluated vaccine batch.

Group 2: Five calves received a subcutaneous injection of six field doses (3 ml each).

Group 3: Five calves received a subcutaneous injection of eight field doses (4 ml each).

Group 4: Five calves received a subcutaneous injection of twelve field doses (6 ml each).

Group 6: Two calves were maintained as the unvaccinated negative control group.

All procedures followed approved protocols, and animals in all groups were housed in separate stables within the CLEVB animal facility. This grouping allowed for assessment of the immune response to varying doses of the vaccine, with comparisons to the unvaccinated control group.

##### Safety

All vaccinated animals, administered with different doses, were kept under clinical observation, and any abnormalities were recorded. Injection sites were carefully examined for adverse effects. In cases where visible or palpable reactions occurred, the exact description of the reaction was noted, as the study groups were not blinded. Additionally, body temperature was measured daily, starting from four days prior to vaccination throughout the vaccine-efficacy study^17^.

##### Serum samples collection

Blood samples were collected at one-week intervals for six weeks from the jugular vein of all animals in all five groups, and sera were separated to conduct the Hemagglutination Inhibition (HI) test and ID Screen ELISA Influenza A Antibody Competition Multi-Species test.

##### Hemagglutination (HA) and hemagglutination inhibition (HI) assays

Hemagglutination (HA) and HI assays were carried out using the standard microtiter plate method as described^18,19^. The HI tests were conducted with 4 HA units of The HPAI H5N1 virus, clade 2.3.4.4b per well.

##### ID screen ELISA influenza A antibody competition multi-species test

All serum samples were tested using the ID Screen^®^ Influenza A Antibody Competition Multi-Species kit (version 0917, Innovative Diagnostics (ID), France). The testing procedure for bovine application followed the guidelines provided in Addendum FLUACA ver 220,424 EN (Related information).

#### Milk antibody transfer

##### Animal group and vaccine dosage

Three lactating native Egyptian cows(Menufi breed)(Group 5) were vaccinated subcutaneously with four field doses (2 ml each) of the H5 AI vaccine, with the dosage determined after an initial four-week antibody screening of the vaccinated calf groups(1–4).

##### Safety

the vaccinated cows (Group 5) were kept under clinical observation.

##### Milk and serum samples collection and testing

Milk and serum samples were collected weekly from the vaccinated cows (Group 5) until antibodies were detected. Milk and serum samples were analyzed using the ID Screen ELISA Influenza A Antibody Competition Multi-Species Test to determine the presence and transfer of antibodies from serum to milk.

### Ethical approval for animal experiments

The current study follows the Animal Research: Reporting of In-Vivo Experiments (ARRIVE) guidelines. All procedures involving animal use strictly adhere to the guidelines established by the Institutional Animal Care and Use Committee at the Agricultural Research Center(ARC-IACUC). Ethical approval for this study was obtained from the committee(ARC-IACUC) approval No ( ARC-CLEVB-98-24). The manuscript is considered compliant with bioethical standards in good faith.

No anesthesia or euthanasia protocols were employed for the animals involved in this study, as all animal-dependent methodological procedures were categorized as either no or low-pain procedures that can be ethically performed on a conscious and alive animal.

### Statistical analysis

The statistical analysis was conducted using Python with SciPy (Version:1.14.0. available at: https://scipy.org/) to assess the differences in hemagglutination inhibition (HI) values among the vaccinated groups (Groups 1 to 4). The Friedman test was employed to determine if there were significant differences in the immune responses measured by HI values across these groups.

## Results

### Evaluation of Humoral Immune Response

#### Safety observations

The commercial The H5 Avian Influenza vaccine batch was evaluated at CLEVB for potency against H5N1 using HI and a challenge test. The vaccine batche demonstrated satisfactory results.

The safety of the inactivated H5 Avian Influenza vaccine was assessed across various dosage groups to evaluate both localized and systemic reactions. Detailed observations include:

##### Groups 1 and 2 (2 mL and 3 mL doses)

Calves receiving these doses exhibited no adverse effects, with normal behavior and feeding patterns throughout the study period.

##### Group 3 (4 mL dose)

**Fig. 1:**
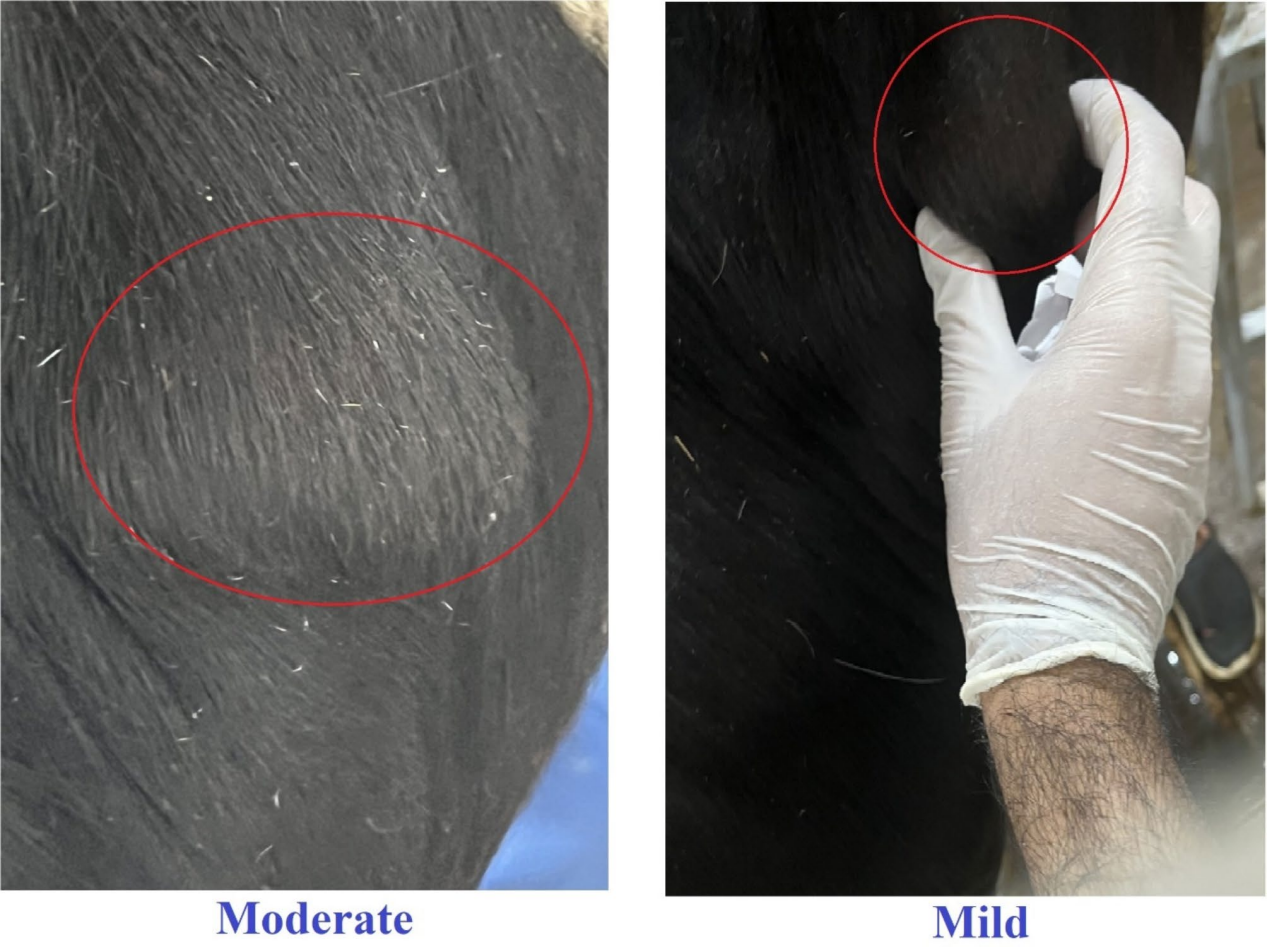
Dose-Dependent Inflammatory Response at the Injection Site Following H5 Avian Influenza Vaccination in Cattle.

Calves in this group experienced mild to moderate aseptic inflammation at the injection site. Swelling was first noticeable by day three post-vaccination and completely resolved within one month without medical intervention, as in shown in Fig. 1.

##### Group 4 (6 mL dose)

Moderate aseptic inflammation, similar to Group 3, was observed. No systemic reactions, such as fever or lethargy, were recorded, as in shown in Fig. 1.

##### Group 6 (control group)

Calves in this unvaccinated group exhibited no adverse reactions, confirming the baseline safety of the study conditions.

These localized inflammatory responses typically appeared around the third day post-vaccination and persisted for approximately one month without requiring treatment. Continuous monitoring ensured the animals’ well-being throughout the study.

#### Humoral immune response

The humoral immune response to the vaccine was analyzed using Hemagglutination Inhibition (HI) and ELISA tests over a six-week period.

#### Hemagglutination inhibition (HI) test

The HI test measured the antibody titers generated in response to vaccination. The results are summarized in Table 1 and show clear dose-dependent responses:

##### Week 1

All vaccinated groups demonstrated measurable antibody responses. Group 1 (2 mL) started with a titer of 4 log_2_, increasing to a peak of 6 log_2_ in Group 4 (6 mL dose). The control group remained at baseline with no detectable antibody titers.

##### Week 2

A significant increase in antibody titers was observed across all vaccinated groups. Group 4 achieved the highest titer of 9 log_2_, while Group 1 reached 6 log_2_.

##### Weeks 3–6

Antibody titers plateaued, with Group 4 maintaining the peak titer of 9 log_2_. Group 1 stabilized at 6 log_2_, while Groups 2 and 3 maintained intermediate titers of 7 and 8 log_2_, respectively. The unvaccinated group showed no response throughout the study.

A statistically significant difference among groups was confirmed using the Friedman test (*p* = 0.0018, χ2 = 15). A summary of the HI titers is provided in Table 1.Friedman Test Statistic (Chi-Square): The Friedman test statistic (chi-square value) of 15 indicates the overall difference among the groups (or conditions) across repeated measures. P-Value: The p-value of 0.0018 suggests that there is a statistically significant difference among the groups, as shown in Table 1.

**Table 1:** Kinetics of Hemagglutination inhibition (HI) antibody response in cattle vaccinated with different doses of inactivated H5 avian influenza vaccine.

Weeks post-vaccination	Mean HI Antibody titer (log_2_)				
	Group (1) 4 × (2 ml)	Group (2) 6 × (3 ml)	Group (3) 8 × (4 ml)	Group (4) 12 × (6 ml)	Group (6) Non vaccinated
1st	*4	5	5	6	0
2nd	6	7	8	9	0
3rd	6	7	8	8	0
4th	6	8	9	9	0
5th	6	8	9	9	0
6th	6	8	9	9	0
**p-value	0.0018				
***χ^2^	15				

*A minimum HI titer of ≥ 5 log_2_ is considered the serological potency standard for protecting birds against mortality, while HI titers ≥ 7 log_2_ can prevent virus shedding from the oropharynx in vaccinated birds.

**p-value ˂ 0.05 is significant.

***The Friedman test statistic (chi-square value) denoted as χ^2^.

#### ELISA test results: serum samples

The ELISA test, conducted using the ID Screen ELISA Influenza A Antibody Competition Multi-Species kit, provided further confirmation of humoral immune responses in serum. The results are detailed in Table 2 and summarized below:

##### Week 1

Group 1 exhibited no detectable antibodies (-), while Groups 2–4 showed increasing levels of positivity (+, ++, +++ respectively).

##### Week 2

All vaccinated groups demonstrated robust antibody responses. Group 1 achieved strong positivity (++), with Group 4 reaching the highest level of positivity (++++) in serum. The control group remained negative (-).

##### Weeks 3–6

Antibody levels plateaued, with Groups 1–4 maintaining their respective positivity levels ( + + to ++++).

### Milk antibody transfer

#### Safety observations

**Group 5** (Lactating cows, 2 mL dose): No visible abnormalities or changes in behavior were noted. Milk production remained consistent and unaffected by vaccination.

#### ELISA test results: milk and serum samples

The ability of the vaccine to induce milk antibody transfer was specifically assessed in Group 5 (lactating cows). Weekly milk amd serum samples analyzed by ELISA showed in Table (2):

Detectable antibodies in Serum samples from Group 5 (lactating cows) mirrored Group 1 results, while milk beginning at week 2, with a strong response (+++) as indicated by ELISA positivity levels.

Antibody levels in milk closely correlated with serum responses, confirming effective systemic and localized humoral immunity.

**Table 2:** H5 avian influenza vaccine: dose-dependent antibody response in cattle serum and milk detected by ID Screen^®^ ELISA test (S/N%).

Weeks post-vaccination			Mean ELISA (S/*N*%)					
	Group (1) 4 × (2 ml)	Group (2) 6 × (3 ml)		Group (3) 8 × (4 ml)	Group (4) 12 × (6 ml)	Group (5) (Serum) 4 × (2 ml)	Group (5) (Milk) 4 × (2 ml)	Group (6) Non vaccinated
1st	-*	+		++	+++	-	±	-
2nd	++	++		++++	++++	++	+++	-
3rd	++	++++		++++	++++	N/A	N/A	-
4th	+++	++++		++++	++++	N/A	N/A	-
5th	+++	++++		++++	++++	N/A	N/A	-
6th	+++	++++		++++	++++	N/A	N/A	-

*Samples presenting a S/N%: - greater than or equal to 50% are considered negative (-).

- Between 45% and 50% are considered doubtful (±). - less than or equal to 45% are considered positive (+).

- (++) ≤ 40–20% - (+++) ≤ 19–10% - (++++) ≤ 9%.

## Discussion

The recent detection of Highly Pathogenic Avian Influenza (HPAI) A(H5N1) in dairy cattle in the United States raises significant concerns regarding potential human exposure to the virus. This development follows several months of documented circulation of HPAI A(H5N1) in avian populations, poultry farms, and various mammalian species, including cattle. The extensive presence of the virus increases the likelihood of human contact, emphasizing the potential severity and zoonotic risk associated with certain HPAI variants. This situation highlights the urgent need for enhanced vigilance and stringent biosecurity measures to prevent the spread of the virus among animals and to safeguard human health^8,10,11,13^. One of the effective strategies for managing Avian Influenza Virus (AIV) in Birds involves vaccination programs, which are pivotal for its control. In Egypt, both monovalent and combined inactivated vaccines against AIV are currently employed to prevent infection^16,20^.

This study aimed to evaluate the efficacy of various doses of an inactivated H5 AI vaccine in cattle, as well as the antibody transmission in milk, against a recent bovine isolate of Highly Pathogenic Avian Influenza (HPAI) A(H5N1, clade 2.3.4.4b). The evaluation was conducted using the Hemagglutination Inhibition (HI) test and ELISA.

Calves from Groups 1 to 4 were subcutaneously inoculated with various doses of the AI vaccine, while the lactating cows in Group 5 were inoculated four weeks post-inoculation of the initial four groups to determine the appropriate dose. In our safety evaluation, the localized reactions observed in Groups 3 (mild to moderate) and 4 (moderate) for 4 ml (8x) and 6 ml (12x), respectively, were consistent with hypersensitivity type III responses, primarily affecting the injection site. Importantly, the administration of the vaccine did not impact milk production in Group 5 cattle (2 ml (4x)), and the rectal temperatures remained within normal ranges in all vaccinated Groups and were comparable to the control values, indicating its safety concerning this critical aspect of cattle farming. In summary, our evaluation supports the overall safety of the inactivated H5 AI vaccine in cattle for doses of 2 ml (4x) and 3 ml (6x) for Groups 1 and 2, respectively, highlighting localized inflammatory responses as the primary concern at higher doses in Groups 3 and 4. These results provide valuable insights for optimizing vaccine formulations and administration protocols in cattle husbandry.

Interestingly, another study in cattle observed a slight increase in body temperatures and local reactions at the inoculation site a few days post-vaccination with their inactivated Lumpy Skin Disease Virus vaccine, which persisted for several days^21^. Another study evaluated the safety of a Newcastle Disease-Avian Influenza (ND-AI) bivalent vaccine in chickens using Marcol white mineral oil adjuvant and Montanide ISA70 adjuvant. The results indicated that both vaccine candidates were free from foreign contaminants and safe for vaccinating chickens, as no detectable signs of infection were observed. Histopathological examinations revealed inflammatory lesions at the injection site. Tissue samples taken on days 3, 7, and 56 post-vaccination showed the presence of local inflammatory lesions, indicating a localized reaction to the vaccine^22^.

In our study assessing the humoral immune response in calves immunized with various doses of an inactivated H5 AI vaccine, hemagglutination inhibition (HI) titers against the AIV clade 2.3.3.4b were measured. The HI titers at one week post-vaccination were 4, 5, 5, and 6 log_2_ for Groups 1, 2, 3, and 4, respectively. These results indicate a robust immune response to the inactivated H5 AI vaccine across all groups, even at the lower dose administered to Group 1 (2 ml 4x). By the fourth week post-vaccination, the HI titers peaked at 8, 9, and 9 log_2_ for Groups 2, 3, and 4, respectively, and remained elevated through the end of the study at six weeks post-vaccination. In contrast, Group 1 exhibited a stable HI titer of 6 log_2_ from the second week through the sixth week post-vaccination.

In a similar study conducted on ducks and chickens using inactivated AI vaccines, it was observed that the ducks developed antibodies by the 3rd week post-vaccination against the H5 virus. The mean HI antibody titers against the H5-Re13 and H5-Re14 viruses ranged from 6.7 log_2_ to 7.2 log_2_. Additionally, the mean HI antibody titers against the DK/FJ/S1424/20 (H5N6) and WS/SX/4 − 1/20 (H5N8) viruses were 6.5 log_2_ and 6.2 log_2_, respectively. On the other hand, the mean HI antibody titers at 3 weeks post-vaccination in chickens against the vaccine strains H5-Re13 and H5-Re14 viruses ranged from 7.5 log_2_ to 8.1 log_2_. Furthermore, the mean HI antibody titers in chickens against the DK/FJ/S1424/20 (H5N6), WS/SX/4 − 1/20 (H5N8), DK/GX/S30428/21 (H5N6), and DK/GD/S4525/21 (H5N1) viruses were 7.3 log_2_, 7.5 log_2_, 6.9 log_2_, and 6.8 log_2_, respectively^23^.

The statistical analysis revealed that the Friedman test statistic was computed as χ2 = 15, with a corresponding p-value of 0.0018. This finding indicates statistically significant differences in HI values among the vaccinated groups (Groups 1 to 4), as detailed in Table 1. The use of Python with SciPy facilitated a robust statistical examination of the HI values, offering valuable insights into the comparative immune responses triggered by various doses of the H5 AI vaccine in cattle. This analysis underscores the importance of dosage in eliciting distinct immune reactions, contributing to the understanding and optimization of vaccine strategies for bovine populations.

The humoral immune response observed was consistent with the ID Screen ELISA Influenza A Antibody Competition Multi-Species results. Initially, Group 1 showed negative results in the first week post-vaccination, whereas Groups 2, 3, and 4 displayed positive results. From the 2nd week through the 6th week post-vaccination, ELISA results indicated persistent and robust antibody presence in Groups 1, 2, 3, and 4, with Group 4 consistently exhibiting the strongest positivity. Although lower vaccine doses provided significant protection, the highest dose was most effective. Non-vaccinated cattle did not develop measurable antibodies against the virus, underscoring the necessity of vaccination. Interestingly, in a similar study, all chickens vaccinated with the inactivated AIV H9N2 vaccine seroconverted within 14 days post-vaccination as confirmed by competitive ELISA (cELISA). Conversely, all non-vaccinated birds remained seronegative. These findings were consistent with the results obtained from the HI test^24^.

In lactating cows (Group 5) inoculated with 2 ml (4x) of inactivated H5 AI vaccine, Milk Antibody Transfer was assessed. Initially, ID Screen ELISA Influenza A Antibody Competition Multi-Species results indicated negative antibody results in serum samples at the 1st week post-vaccination, while milk samples showed doubtful results. By the 2nd week post-vaccination, serum samples from Group 5 tested positive, whereas milk samples exhibited a strong positive response in ID ELISA testing. Our findings clearly demonstrated that the ID ELISA test had higher sensitivity for detecting antibodies against AIV in milk samples compared to serum samples. This discrepancy may be attributed to the larger volume of milk samples used in ID ELISA, as recommended by guidelines provided in Addendum FLUACA ver 220,424 EN (related information), potentially reducing troubleshooting and interference issues observed with serum samples. In a 2014 study, a high level of agreement (94.4%) and concordance (kappa = 0.865) was reported between 125 paired serum and individual milk samples. The study suggested that individual milk could serve as a viable alternative to blood collection and serum testing^25^.

Regarding AIV in cattle, no studies have yet established the duration for which antibodies can be detected in milk after natural infection or post-vaccination. Consequently, the results reported in this study should be considered in light of these limitations.

The limitations of this study can be addressed by noting that the humoral immune response and Milk Antibody Transfer were assessed using an AIV vaccine designed for birds. Various doses of the AI vaccine were administered based on the recommended chicken doses, and the number of animals in each group was sufficient for statistical analysis. A larger sample size would provide more reliable data. Nonetheless, the study proceeded with a carefully planned duration and well-managed sampling intervals. This step is crucial to establish the feasibility of the immune response of cattle to the current AI vaccine. To enhance the precision of the study, future investigations should delve deeper into factors influencing the formulation of AI vaccines for cattle, such as adjuvant and antigen content. Understanding and addressing these variables will contribute to a more accurate assessment of bovine immune response and immune receptors. Additionally, extending the monitoring period post-vaccination, increasing the sample size, and exploring different vaccine formulations could provide more comprehensive insights into the efficacy and longevity of the immune response in cattle. These efforts will help optimize vaccine strategies for bovine populations, ensuring better protection against AIV. By addressing these limitations and refining the study parameters, we can improve our understanding of the immune response in cattle to AI vaccines and develop more effective vaccination protocols tailored to bovine needs.

The study revealed a clear dose-dependent pattern in the cattle’s immune response to the H5 avian influenza vaccine, with higher doses resulting in stronger and more sustained antibody responses. Even the lower doses provided significant antibody protection, achieving titers within the protective range of 5 to 7 log_2_, as established in chickens. A minimum hemagglutination inhibition (HI) titer of ≥ 5 log_2_ has been suggested as a serological potency standard indicative of protection, safeguarding birds against mortality. Furthermore, HI titers of ≥ 7 log_2_ have been associated with the prevention of virus shedding from the oropharynx in vaccinated birds^19,26^. This response was corroborated by the ID Screen ELISA results. Notably, the humoral immune response in all vaccinated groups elicited a rapid and high antibody response within the early weeks post-vaccination. This response stabilized quickly and persisted consistently throughout the experimental period.

However, the cattle’s immune response to the H5 AI vaccine differed markedly from their responses to other vaccines, such as those for Foot and Mouth Disease Virus (FMDV)^27–29^ and Lumpy Skin Disease Virus (LSDV)^30^. Several factors could explain these differences. The adjuvant and antigen composition of the H5 AI vaccine may be particularly effective in inducing a strong and stable immune response in cattle, unlike the formulations used for FMDV and LSDV vaccines. Additionally, the H5 AI vaccine may possess higher immunogenicity in cattle compared to the FMDV and LSDV vaccines, leading to a more robust production of antibodies. The nature of the H5 avian influenza virus itself may trigger a different immune mechanism in cattle^31^, resulting in a distinct and more effective antibody response compared to other viruses. Furthermore, the observation of a dose-dependent response underscores the importance of optimizing vaccine dosage, as higher doses of the H5 AI vaccine achieved better immune responses, a factor that might need consideration for other vaccines. Finally, the rapid stabilization and persistence of the antibody response to the H5 AI vaccine suggest that the immune system of cattle responds more favorably to this particular vaccine’s stimulation.

## Conclusion

The study demonstrated that the H5 avian influenza vaccine induces a robust, dose-dependent immune response in cattle, with higher doses yielding stronger and more sustained antibody production. The findings underscore the importance of optimizing vaccine dosage and formulation to enhance immune efficacy. The rapid stabilization and persistence of the immune response highlight the vaccine’s potential effectiveness in cattle, a response distinct from that elicited by other vaccines used in cattle. Additionally, the study observed effective milk antibody transfer in lactating cows, indicating that the vaccine not only elicits a systemic immune response but also facilitates the transfer of antibodies through milk. Future research should focus on refining vaccine formulations, considering adjuvant and antigen content, extending the monitoring period, and further investigating milk antibody transfer to optimize the immune response in bovine populations. These efforts are crucial for developing effective vaccination strategies to safeguard cattle against avian influenza and potentially other infectious diseases.

## Data Availability

All data generated or analyzed during this study are included in this published article and related information.
